# Development and validation of a clinical prediction model for prognostic factors in patients with primary pontine hemorrhage

**DOI:** 10.1590/1414-431X2024e13359

**Published:** 2024-04-19

**Authors:** Anquan Hu, Heyan Qin, Shina Wu, Xiaolin Zhao, Yumeng Li, Feng Chen, Tao Liu

**Affiliations:** 1 Department of Geriatric Center, Hainan General Hospital (Hainan Affiliated Hospital of Hainan Medical University), Haikou, China; 2 Department of Neurology, Hainan General Hospital (Hainan Affiliated Hospital of Hainan Medical University), Haikou, China; 3 Department of Neurology, Nanfang Hospital, Southern Medical University, Guangzhou, China; 4 Department of Radiology, Hainan General Hospital (Hainan Affiliated Hospital of Hainan Medical University), Haikou, China

**Keywords:** Primary pontine hemorrhage, Nomogram, Predictive model, Outcome

## Abstract

We aimed to develop a prognostic model for primary pontine hemorrhage (PPH) patients and validate the predictive value of the model for a good prognosis at 90 days. A total of 254 PPH patients were included for screening of the independent predictors of prognosis, and data were analyzed by univariate and multivariable logistic regression tests. The cases were then divided into training cohort (n=219) and validation cohort (n=35) based on the two centers. A nomogram was developed using independent predictors from the training cohort to predict the 90-day good outcome and was validated from the validation cohort. Glasgow Coma Scale score, normalized pixels (used to describe bleeding volume), and mechanical ventilation were significant predictors of a good outcome of PPH at 90 days in the training cohort (all P<0.05). The U test showed no statistical difference (P=0.892) between the training cohort and the validation cohort, suggesting the model fitted well. The new model showed good discrimination (area under the curve=0.833). The decision curve analysis of the nomogram of the training cohort indicated a great net benefit. The PPH nomogram comprising the Glasgow Coma Scale score, normalized pixels, and mechanical ventilation may facilitate predicting a 90-day good outcome.

## Introduction

Primary pontine hemorrhage (PPH) accounts for approximately 5-10% of all intracranial hemorrhages and is characterized by a varied prognosis (1). Untraceable PPH, often linked to hypertensive disease, is a severe form of spontaneous cerebral hemorrhage with an acute mortality rate ranging from 40 to 60% (2). The prognosis of PPH is influenced by various clinical characteristics and the extent of bleeding. Consequently, several grading scales have been devised to assess PPH, including the intracerebral hemorrhage (ICH) score, the PPH grading scale, the New PPH grading scale, and the intracerebral hemorrhage scale (3-5). The ICH score indicates that sub-tentorial cerebral hemorrhage is a critical factor contributing to the unfavorable prognosis of ICH. Given the limited size of the pontine structure, an equivalent extent of bleeding in the pontine region may yield more severe symptoms and a worse prognosis than a supratentorial cerebral hemorrhage. Consequently, the established cutoff values and scoring guidelines within the ICH score for supratentorial cerebral hemorrhage may not be entirely applicable to PPH.

The aforementioned scales are employed for the prediction of patient mortality and prognosis based on bleeding volume, which is currently measured manually, resulting in significant inconvenience and inaccuracy. The identification of prognostic factors is of paramount importance for the formulation of a rational diagnosis and treatment strategy, allocation of medical resources, and effective patient management. The prognostic implications of various clinical and computed tomography (CT) parameters have been characterized in patients with PPH, but appropriate treatment options for severe PPH, which are determined based on these parameters, have not yet been developed (4). Currently, there is a lack of a widely accepted early prognostic model for predicting outcomes in patients with PPH. Our objective was to develop a novel clinical nomogram model that accurately predicts the likelihood of a good outcome within 90 days for PPH patients.

Nomogram is a clinical tool that visually displays a statistical model and provides a numerical probability of the target event (6). In this study, we reviewed long-term survival data from a large cohort to identify risk factors and establish predictive nomogram models. We also aimed to validate the predictive value of these nomogram models for 90-day good outcomes in PPH patients.

## Material and Methods

### Study design and participants

**Figure 1:**
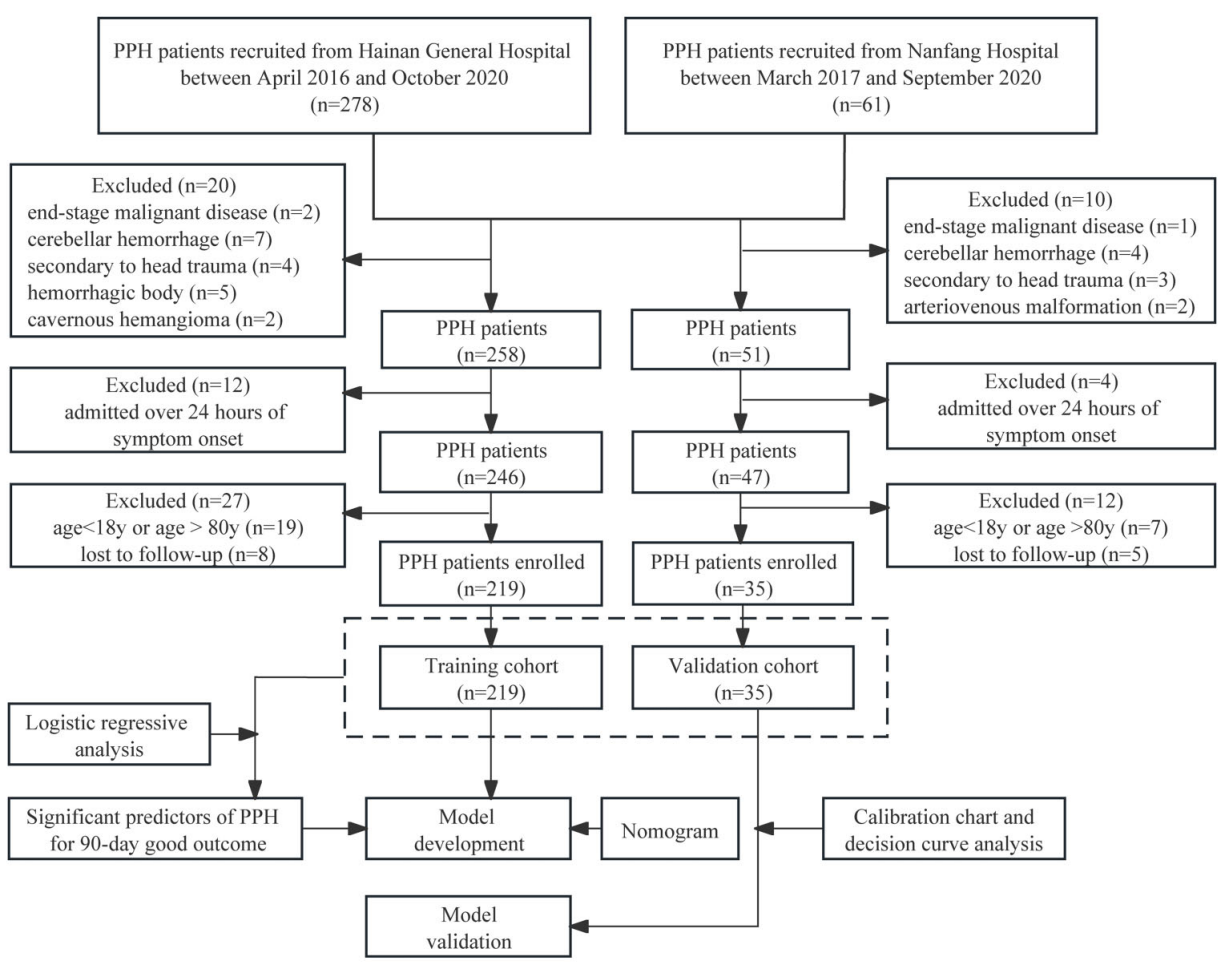
Flow diagram of study selection. PPH: primary pontine hemorrhage.

In this study, we collected datasets from 2 centers for screening independent predictors. A total of 278 PPH patients were screened from Hainan General Hospital between April 2016 and October 2020 using the inclusion and exclusion criteria cited below, and 219 PPH patients were enrolled in the study. A total of 61 PPH patients were screened from Nanfang Hospital between March 2017 and September 2020, and 35 PPH patients were enrolled in the study. The 219 PPH patients enrolled from Hainan General Hospital were used as the training cohort, and the 35 PPH patients enrolled from Nanfang Hospital were used as the external validation cohort. Figure 1 illustrates the flow of patient recruitment and the model for prognostic prediction of PPH. This study was approved by the Medical Ethics Committee of Hainan General Hospital (ChiCTR2100042705; http://www.chictr.org.cn). All patients were screened consecutively based on the inclusion criteria: 1) PPH diagnosed by CT imaging and admitted within 24 h of symptom onset; 2) Patients between the ages of 18 and 80 years; 3) Completion of a cranial CT scan with the retrieval of Digital Imaging and Communications in Medicine (DICOM) images; 4) Patients were followed-up regularly for at least 90 days with a functional score on day 90. Exclusion criteria were patients with end-stage malignant disease, cerebellar hemorrhage secondary to head trauma, hemorrhagic body, cavernous hemangioma, or arteriovenous malformation.

### Baseline data and prognostic information

#### Data collection

Data was recorded in a case report form including age, sex, history of hypertension, history of diabetes, history of smoking, alcoholism, need for mechanical ventilation, CT-guided stereotactic hematoma aspiration, extra ventricular drainage, blood glucose, body temperature, systolic blood pressure, diastolic blood pressure, mean arterial pressure, heart rate, respiratory rate, white blood cell count, hemoglobin, platelets, hematocrit, prothrombin time (PT), activated partial thromboplastin time (APTT), blood urea nitrogen (BUN), creatinine (Cr), and Glasgow Coma Scale (GCS) score.

#### Calculation of hematoma volume

Head CT DICOM images of each patient were imported into the ITK-SNAP software (USA). The main function of ITK-SNAP is to segment medical images, including 2D and 3D segmentation, manual segmentation, and semi-automatic segmentation. The bleeding boundaries of the opened PPH images were manually calibrated layer by layer in the ITK-SNAP software to generate regions of interest (ROI) for bleeding volume calculation. The number of pixels was automatically read by the software and was used as a representation of bleeding volume. We named the number of pixels as normalized pixels (used to describe bleeding volume). We have abandoned the traditional ABC/2 manual calculation mode for the calculation of bleeding volume and adjusted it to manually draw the boundaries of each layer of bleeding on the baseline CT image. The software machine read the number of pixels in all ROI to calculate the bleeding volume.

We used 30- and 90-day mortality and 90-day good outcome as the primary end-point of this study. Functional outcome was measured by the modified Rankin Scale (mRS), where a score between 0 and 3 was defined as a good outcome, and a score between 4 and 6 was defined as a poor outcome (7). Information on survival and outcome was obtained from family members or patients by professionally trained attending physicians of neurology using a blinded telephone call-back method.

### Development of the nomogram model for prognostic analysis of PPH

The training cohort and validation cohort were combined. According to good and poor 90-day functional outcomes, we performed multivariable logistic regression analysis on 26 candidate variables and screened the key factors affecting prognosis. These key prognostic predictors were then selected to construct a nomogram model to predict the 90-day good outcome from the training cohort and validated its efficacy in the validation cohort. The three models were established using GCS scoring, normalized pixels, and a combination of the three metrics named the hybrid model (including GCS score, normalized pixels, and mechanical ventilation).

### Statistical analyses

Continuous variables (mean±SD) were assessed using Mann-Whitney U test or two independent samples *t*-tests. Categorical variables were analyzed using Fisher exact tests or the chi-squared test. Initial screening of potential covariates was based on single factor analysis, and covariates with P<0.05 were employed to perform the multivariate logistic regression analysis.

Significant variables were used to produce the risk model. Receiver operating curves (ROCs) were used to assess the predictive model's discrimination capability for the 90-day good outcome and 30- and 90-day mortality. The specificity, sensitivity, accuracy, and area under the curve (AUC) of each model were calculated. The AUCs were compared using Delong's test.

A nomogram was established according to the results of the multivariable logistic regression to evaluate the risk of 90-day good outcome. The discrimination, calibration ability, and clinical usefulness were used to evaluate the model's performance. The discriminative abilities of nomograms were quantified by ROC curve and concordance index (C-index) measures. The calibration curve was used to determine the calibration ability of the model. Decision curve analysis (DCA) was executed to assess the clinical utility of both nomograms by quantifying the net benefits for a range of threshold probabilities. To evaluate the clinical efficacy of the predictive model, decision curves were analyzed compared to two default strategies: “treat all” and “treat none”. The “treat all” approach assumes all patients are treated regardless of their risk estimates, while the “treat none” strategy assumes none of the patients are at risk and thus not treated. A model demonstrating clinical utility would be positioned above the horizontal “treat none” line and to the right of the downward-sloping “treat all” line. P<0.05 was indicative of a statistically significant difference. All statistical tests were performed using R software (version 4.2.2: https://www.r-project.org/).

## Results

### Patient baseline data

A total of 254 patients who met the criteria were included in the study. A total of 26 variables, encompassing demographic, clinical, radiological, and laboratory information, were employed to assess disparities in outcomes between patients who experienced good and poor outcomes at 90 days. Out of the total, 56.3% (143/254) had a poor outcome, whereas 43.7% (111/254) had a good outcome. According to the findings of our research, in-hospital mortality rate was 36.2% (92/254), of which 70.7% (65/92) of the deaths were attributed to neurological injury. Among them, 62 cases resulted in cardiopulmonary arrest due to the initial neurological damage, and 3 cases expired due to central nervous system infection caused by ventriculitis after the insertion of an external ventricular drainage system. Twenty-nine percent (27/92) of deaths were due to non-neurological injuries. Specifically, 5 cases were attributed to aspiration pneumonia, 5 cases expired due to hospital-acquired infections, 3 cases were caused by acute myocardial infarction, 3 cases were due to gastrointestinal bleeding, and 3 cases were caused by pulmonary thromboembolism. Furthermore, 6 cases were associated with multiple organ dysfunction syndrome, while the cause of death remained unknown in 2 cases.

The univariate analysis revealed a significant correlation between the 90-day good outcome and GCS score, normalized pixels, sex, platelets, and mechanical ventilation (P<0.05, Table 1). Other variables, such as age, history of hypertension, history of diabetes, history of smoking, alcoholism, CT-guided stereotactic hematoma aspiration, extra ventricular drainage, blood glucose, body temperature, systolic blood pressure, diastolic blood pressure, mean arterial pressure, heart rate, respiratory rate, white blood cell count, hemoglobin, hematocrit, PT, APTT, BUN, and Cr, were not found to be statistically significant (P>0.05) (Table 1).

**Table 1:** Baseline characteristics of the 254 patients divided into good or poor outcome based on the modified Rankin Scale (mRS) scores.

Characteristic	4-6=Poor outcome (n=143)	1-3=Good outcome (n=111)	P-value
GCS score	5 (3, 6)	13 (9, 15)	<0.001
Normalized pixels	30,613 (17,242; 46,922)	7,355 (3,582; 16,349)	<0.001
Gender			0.034
Male	121 (85%)	82 (74%)	
Female	22 (15%)	29 (26%)	
Age	53±12	52±13	0.689
Hypertension	107 (75%)	83 (75%)	0.993
Diabetes mellitus	11 (7.7%)	8 (7.2%)	0.884
Smoking	25 (17%)	26 (23%)	0.241
Alcohol abuse	35 (24%)	23 (21%)	0.479
Temperature	37.20 (36.75; 38.00)	37.00 (36.65; 37.50)	0.255
Heart rate	90 (80; 112)	84 (78; 116)	0.210
SBP	177 (155; 191)	174 (156; 190)	0.836
DBP	100 (90; 110)	101 (95; 118)	0.144
MAP	130 (116; 144)	131 (123; 150)	0.220
Respiratory rate	20.0 (18.0; 23.0)	20.0 (18.0; 22.0)	0.961
Glucose level	7.4 (6.3; 8.9)	7.3 (6.2; 8.8)	0.700
White cell count	11.3 (9.0; 13.9)	10.5 (7.8; 14.1)	0.417
Hemoglobin	143 (136; 154)	141 (130; 150)	0.183
Platelet	211 (177; 245)	199 (163; 237)	0.073
Hematocrit	43.1 (41.0; 44.5)	42.0 (39.7; 44.7)	0.197
PT	13.00 (11.90; 14.10)	12.80 (11.40; 13.70)	0.319
APTT	31.0 (26.6; 34.0)	31.0 (26.6; 33.7)	0.431
Creatinine	89 (68; 108)	89 (64; 108)	0.615
Urea	5.8 (4.5; 7.2)	5.5 (4.1; 7.2)	0.378
Mechanical ventilation	57 (40%)	25 (23%)	0.003
Stereo orientation	5 (3.5%)	6 (5.4%)	0.541
Extra ventricular drainage	17 (12%)	6 (5.4%)	0.074

Data are reported as median (IQR), n (%), or mean±SD and were compared using Wilcoxon rank sum test, Pearson's chi-squared test, Welch two independent samples *t*-test, or Fisher's exact test. GCS: Glasgow Coma Scale; SBP: systolic blood pressure; DBP: diastolic blood pressure; MAP: mean arterial pressure; PT: prothrombin time; APTT: activated partial thromboplastin time.

### Key prognostic predictors

GCS score, normalized pixels, sex, platelets, and mechanical ventilation were used as covariates in the multivariate logistic regression analysis. Significant odds ratios were identified for GCS score [1.53 (1.36-1.73), P<0.001], normalized pixels [0.999957 (0.999938-0.999974), P<0.001], and mechanical ventilation [0.13 (0.04-0.39), P<0.001)]. However, there were no significant associations observed for sex [0.74 (0.27-1.95), P=0.547] and platelets [1.00 (0.99-1.00), P=0.383] (Table 2). The prognostic model to predict a 90-day good outcome was based on the GCS score, normalized pixels, and mechanical ventilation.

**Table 2:** Multivariable analysis of characteristics of 254 patients divided into good or poor outcome based on the modified Rankin Scale (mRS) scores.

Characteristic	4-6=Poor outcome, n=143	1-3=Good outcome, n=111	OR	95%CI	P-value
GCS score	5 (3; 6)	13 (9; 15)	1.53	1.37; 1.75	<0.001
Normalized pixels	30,613 (17,242; 46,922)	7,355 (3,582; 16,349)	0.999957	0.999938; 0.999974	<0.001
Gender			0.74	0.27; 1.95	0.547
Male	121 (85%)	82 (74%)			
Female	22 (15%)	29 (26%)			
Age	53±12	52±13	1.0	0.96; 1.03	0.765
Hypertension			1.03	0.37; 2.86	0.958
No	36 (25%)	28 (25%)			
Yes	107 (75%)	83 (75%)			
Diabetes mellitus			0.44	0.08; 2.13	0.322
No	132 (92.3%)	103 (92.8%)			
Yes	11 (7.7%)	8 (7.2%)			
Smoking			1.44	0.49; 4.17	0.500
No	118 (83%)	85 (77%)			
Yes	25 (17%)	26 (23%)			
Alcohol abuse			0.88	0.31; 2.46	0.810
No	108 (76%)	88 (79%)			
Yes	35 (24%)	23 (21%)			
Temperature	37.20 (36.75; 38.00)	37.00 (36.65; 37.50)	0.73	0.51; 1.03	0.075
Heart rate	90 (80; 112)	84 (78; 116)	1.01	0.99; 1.03	0.467
SBP	177 (155; 191)	174 (156; 190)	0.98	0.96; 1.00	0.136
DBP	100 (90; 110)	101 (95; 118)	1.00	0.96; 1.03	0.817
MAP	130 (116; 144)	131 (123; 150)	1.02	0.98; 1.07	0.277
Respiratory rate	20.0 (18.0; 23.0)	20.0 (18.0; 22.0)	0.95	0.88; 1.02	0.158
Glucose level	7.4 (6.3; 8.9)	7.3 (6.2; 8.8)	1.05	0.93; 1.19	0.469
White cell count	11.3 (9.0; 13.9)	10.5 (7.8; 14.1)	1.00	0.88; 1.13	0.939
Hemoglobin	143 (136; 154)	141 (130; 150)	1.00	0.98; 1.02	0.889
Platelet	211 (177; 245)	199 (163; 237)	1.00	0.99; 1.00	0.383
Hematocrit	43.1 (41.0; 44.5)	42.0 (39.7; 44.7)	0.91	0.79; 1.05	0.194
PT	13.00 (11.90; 14.10)	12.80 (11.40; 13.70)	0.96	0.81; 1.14	0.658
APTT	31.0 (26.6; 34.0)	31.0 (26.6; 33.7)	1.01	0.92; 1.11	0.752
Creatinine	89 (68; 108)	89 (64; 108)	1.00	1.00; 1.00	0.784
Urea	5.8 (4.5; 7.2)	5.5 (4.1; 7.2)	0.96	0.86; 1.07	0.424
Mechanical ventilation			0.13	0.04; 0.39	<0.001
No	86 (60%)	86 (77%)			
Yes	57 (40%)	25 (23%)			
Stereo orientation			0.83	0.11; 7.43	0.860
No	138 (96.5%)	105 (94.6%)			
Yes	5 (3.5%)	6 (5.4%)			
Extra ventricular drainage			1.34	0.23; 6.99	0.730
No	126 (88%)	105 (94.6%)			
Yes	17 (12%)	6 (5.4%)			

Data are reported as median (IQR), n (%), or mean±SD. OR: odds ratio; CI: confidence interval.

**Figure 2:**
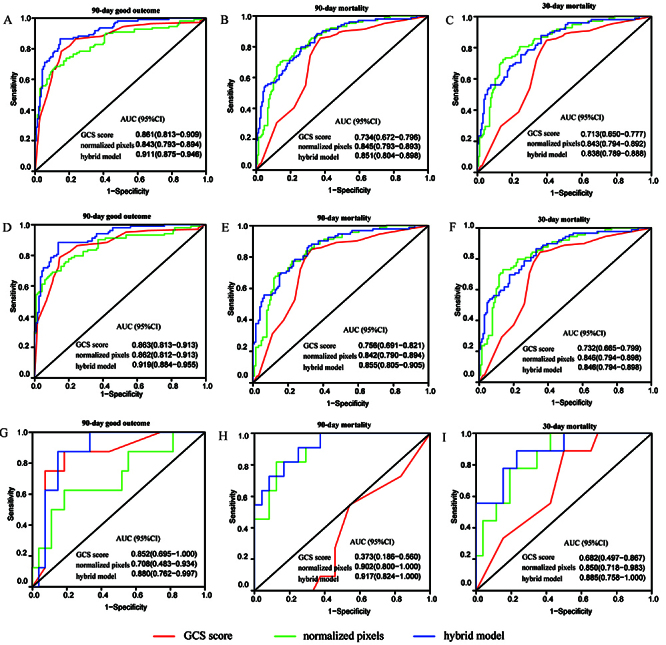
Receiver operating curves (ROC) and area under the curve (AUC) of the Glasgow Coma Scale (GCS) score, normalized pixels, and hybrid model for predicting the 90-day good outcome, and 90- and 30-day mortality in total cases (**A**, **B**, **C**), in the training cohort (**D**, **E**, **F**), and in the validation cohort (**G**, **H**, **I**).

The AUCs of the three predictors for the 90-day good outcome, 90-, and 30-day mortality across all cases are illustrated in Figure 2A, B, and C. The hybrid model achieved the best discrimination ability for the 90-day good outcome [AUC of hybrid model 0.911 (95%CI: 0.875−0.946), hybrid model *vs* GCS score, P=0.001, hybrid model *vs* normalized pixels, P=0.006] and 90-day mortality [AUC of hybrid model 0.851 (95%CI: 0.804−0.898), hybrid model *vs* normalized pixels, P<0.001].

The AUCs of the three predictors for the 90-day good outcome, 90-, and 30-day mortality in the training cohort are illustrated in Figure 2D, E, and F. The hybrid model achieved the best discrimination ability for the 90-day good outcome [AUC of hybrid model 0.863 (95%CI: 0.813−0.913), hybrid model *vs* GCS score, P<0.001, hybrid model *vs* normalized pixels, P=0.02] and 90-day mortality [AUC of hybrid model 0.855 (95%CI: 0.805−0.905), hybrid model *vs* normalized pixels, P=0.006, hybrid model *vs* GCS score, P=0.5], as well as in 30-day mortality [AUC of hybrid model 0.846 (95%CI: 0.794−0.898), hybrid model *vs* GCS score, P<0.001, hybrid model *vs* normalized pixels, P=1].

The AUCs of the three predictors for 90-day good outcome, 90-, and 30-day mortality in the validation cohort are illustrated in Figure 2G, H, and I. The hybrid model achieved the best discrimination ability for the 90-day mortality [AUC of hybrid model 0.917 (95%CI: 0.824−1.000), hybrid model *vs* GCS score, P<0.001, hybrid model *vs* normalized pixels, P=0.6] and 30-day mortality [AUC of hybrid model 0.885 (95%CI: 0.758−1.000), hybrid model *vs* GCS score, P=0.03, hybrid model *vs* normalized pixels, P=0.5]. The hybrid model had no discrimination ability for the 90-day good outcome [AUC of hybrid model 0.880 (95%CI: 0.762−0.997), hybrid model *vs* GCS score, P=0.5, hybrid model *vs* normalized pixels, P=0.1].

### Prognostic performance of the nomogram model

**Figure 3:**
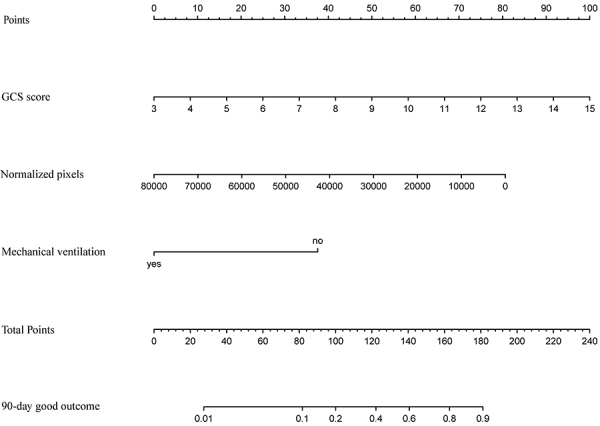
Nomogram predictive model for the 90-day good outcome. The GCS score, normalized pixels, and mechanical ventilation factors were included in the nomogram. GCS score: A higher GCS score was generally associated with a better prognosis, indicating a higher level of consciousness. Normalized pixels: Lower values of normalized pixels suggested a smaller volume of hemorrhage, which was typically linked to a better outcome. Mechanical ventilation: The patients without mechanical ventilation had a better prognosis. The total score, derived from adding the individual points from the GCS score, normalized pixels, and mechanical ventilation, was used to estimate the probability of a 90-day good outcome.

The GCS score, normalized pixels, and mechanical ventilation factors were included in the model, fitted, and represented as a nomogram (Figure 3). While using this nomogram, each patient predictor was located on the corresponding axis. A line was then drawn on the top score axis to generate a score based on each variable. Finally, the scores from all variables were added to calculate the total score. This was located on the “total score” axis, and a straight line was drawn down to generate the nomogram for the 90-day good outcome of the patient in the training cohort.

**Figure 4:**
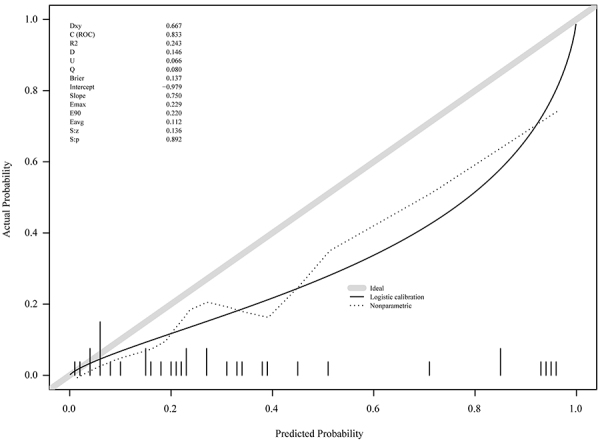
Calibration chart between the training cohort and the validation cohort. The calibration chart showed that the observed and predicted values were consistent in the training cohort, with no statistical difference (P=0.892) between the training cohort and validation cohort, indicating a good fit. The new model showed strong discrimination ability [C index (AUC)=0.833].

The calibration chart indicated that the observed and predicted values were consistent in the training cohort, and the U test showed no statistical difference (P*=*0.892) between the training cohort and the validation cohort, suggesting that the model fitted well (Figure 4). The new model also showed good discrimination ability [C index (AUC)=0.833] (Figure 4). The threshold probability is plotted on the x-axis, and the net benefit of the nomogram is plotted on the y-axis.

**Figure 5:**
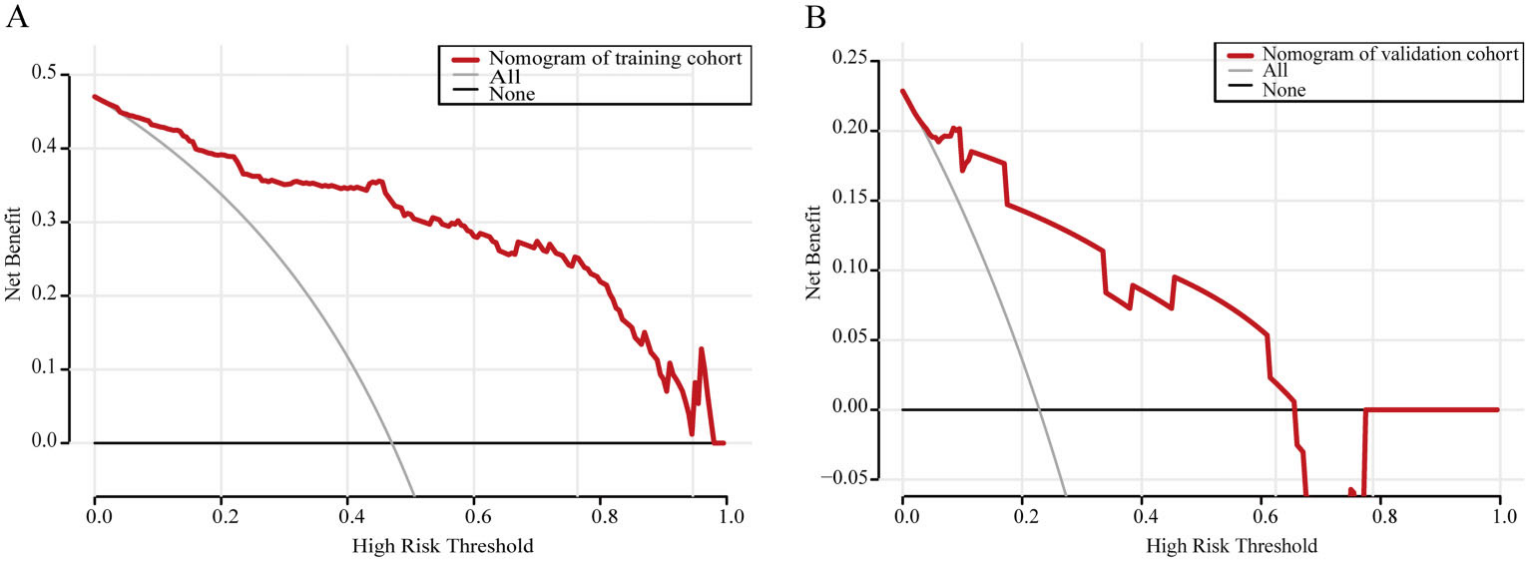
Decision curve analysis of our nomogram in the training cohort (**A**) and validation cohort (**B**).

The DCA showed a greater net benefit for the nomogram than for “All” or “None” scheme across a reasonable range of threshold probability. The DCA of the training cohort nomogram indicated a great net benefit when the threshold probability was from 0.5 to 97.5% (Figure 5A). Similarly, the DCA of the validation cohort nomogram indicated a great net benefit when the threshold probability was from 2.5 to 66.5% (Figure 5B).

## Discussion

This study aimed to develop a nomogram for 90-day good outcome prognostic factors in patients with PPH using relatively large samples. A total of 254 patients with PPH were included in the study, with 219 patients from the training cohort and 35 patients from the validation cohort. PPH is the deadliest type of ICH. Our study found an in-hospital mortality rate of 36.2% (92/254), of which 70.7% (65/92) of deaths were caused by initial neurological damage resulting from cardiopulmonary arrest. In another study, the in-hospital case fatality rate was 47.5% (8). Our study indicated that GCS score, normalized pixels, and mechanical ventilation were optimal combinations for predicting 30-, 90-day mortality, and 90-day good outcome.

GCS score, normalized pixels, and mechanical ventilation were significant predictors of 90-day good outcome in PPH. Fu et al. (9) found that GCS score and hematoma locations were independently associated with severity on admission and in-hospital mortality after primary intracerebral hemorrhage. Wessels et al. (10) found a high correlation between a poor outcome (GCS score <4) and hematoma volume greater than 4 mL (P*=*0.006), ventral hemorrhage (P<0.001), and necessity for mechanical ventilation (P<0.001). Zhang et al. (11) found that GCS score on admission and coma were the only significant predictors of mortality with multivariate regression analysis. Consistent with other studies, as one of the most important predictors for intracerebral hemorrhage prognosis models, GCS score also showed a good predictive performance in PPH, especially in predicting 90-day good outcome in our study, with AUC values of 0.861, 0.863, and 0.852 in all cases, the training cohort, and the validation cohort, respectively.

Hemorrhage volume is also one of the important factors in predicting the prognosis of intracerebral hemorrhage. The formula ABC/2 is widely used to calculate cerebral hemorrhage volume (12). However, the manual measurement methods can be susceptible to human error. Mishra et al. (13) found that the measurement of intracerebral hemorrhage volume using ITK-SNAP had better reliability compared to the manual ABC/2 method when assessed using inter-observer reliability statistics. We used the number of pixels (normalized pixels) automatically read by the ITK-SNAP software as a representation of bleeding volume. In our research, normalized pixels [min=505, max=282005, median (IQR)=17505 (5794, 37421)] represented bleeding volume. It replaced bleeding volume more accurately and demonstrated good predictive performance (the AUC values in predicting 90-day good outcome were 0.843, 0.862, and 0.708 in all cases, the training cohort, and the validation cohort, respectively). Kuwabara et al. (14) reported that cases with maximum hematoma diameters of 20 mm or less and seen in three CT slices or less of 10 mm thickness have hematoma confined to the pons with favorable prognoses. Our cases also suggested that a major bleeding tends to have a worse prognosis, while a small bleeding has a better prognosis, some of which manifest as hemorrhagic lacunar stroke, with sizes ranging from 0.5 to 1.8 mL with a mean of 1.19 mL in our cases. Arboix et al. (15) found that hemorrhagic lacunar stroke accounted for 7.4% of intracerebral hemorrhages. The patients with hemorrhagic lacunar stroke showed a higher percentage of symptom-free patients at the time of discharge and absence of in-hospital mortality. The proportion of hemorrhagic lacunar stroke in our study was 9.8%, the overall prognosis was good, and there were no deaths. This may be related to the smaller size of the lesion in hemorrhagic lacunar stroke (15).

Mechanical ventilation was a risk factor for mortality and 90-day good outcome in patients of PPH. Thirty-two percent of patients used mechanical ventilation in our research, which was consistent with reports in the literature (16,17). Previous studies found that signs of brainstem dysfunction predicted higher mortality for intracerebral hemorrhage and cerebral infarction (17). Zou et al. (18) found that mechanical ventilation is a risk factor in patients with intracerebral hemorrhage, and 56.91% patients used mechanical ventilation in their study. The utilization rate of mechanical ventilation in their study was higher than ours. This difference can be attributed to the fact that their research subjects consisted solely of patients in the intensive care unit, excluding those with mild symptoms.

ROC curves showed how well a risk prediction model discriminates between patients with and without a condition (19). Xie et al. (20) found that the hybrid model achieved satisfactory discrimination. In our study, the AUC of the hybrid model of combined normalized pixels and GCS score was 0.919, the AUC of GCS score was 0.863, and the AUC of normalized pixels was 0.862 for the 90-day good outcome in all cases. It indicated that the hybrid model had a better predictive ability than the GCS score or normalized pixels alone in predicting 90-day good outcome. However, concerning predicting 30- and 90-day mortality, the hybrid model did not outperform the model with normalized pixels alone. Of course, this requires a larger sample size for further validation. The nomogram including the GCS score, normalized pixels, and mechanical ventilation was constructed for predicting the 90-day good outcome. The nomogram revealed that a higher GCS score was associated with a better patient prognosis, indicating it as a protective factor, which was consistent with previous research (3). The smaller the assigned score, the more likely is the patient to show a 90-day good outcome. A smaller value of normalized pixels indicates a better prognosis. Compared to the New PPH score (3), our method had a more refined division of bleeding volume. Mechanical ventilation is a binary variable, and not requiring mechanical ventilation is associated with a better prognosis for the patient. The GCS score had the strongest impact on prognosis in this study, which aligned with the findings of the ROC curve. The predicted probability value of the 90-day good outcome is determined by the percentage ratio corresponding to the total score of these three factors. External validation further supported the reliability of the model. The DCA of the nomogram in the training cohort and validation cohort showed a net benefit for predicting a 90-day good outcome, surpassing both the treat-all and treat-none strategies. These findings highlight the strong clinical applicability of our predictive model.

Our study had several limitations. Firstly, although the model was externally validated and the nomogram model achieved good accuracy, there is still a need for further prospective multicenter validation to confirm and enhance the reliability of the nomogram and improve its clinical utility. Secondly, the number of pixels did not provide an intuitive indication of bleeding volume. In future stages, we aim to develop software or programs that can automatically convert pixels into bleeding volume measured in milliliters (mL).

Future research may explore two main directions. Firstly, we can focus on the application of multimodal imaging technology, such as functional magnetic resonance imaging (fMRI), computed tomography (CT), and magnetic resonance imaging (MRI). By integrating these technologies, we can accurately identify the bleeding site and assess the extent of brain tissue damage. Secondly, we will investigate molecules and biomarkers in blood and brain tissues to gain a deeper understanding of the pathological mechanisms of cerebral hemorrhage. Inflammatory factors, coagulation factors, and metabolites are among the biomarkers that could be studied. These biomarkers hold significant potential for predicting the risk and prognosis of cerebral hemorrhage.

### Conclusion

In this study, we developed and validated a nomogram comprising the GCS score, normalized pixels, and mechanical ventilation for predicting the 90-day good outcome in PPH patients. Our model showed strong discriminatory power, calibration, and clinical utility, allowing doctors to predict the survival of patients with PPH conveniently and quickly.
